# Characterisation of household single-use packaging flows through a municipal waste system: A material flow analysis for New South Wales, Australia

**DOI:** 10.1016/j.heliyon.2024.e32878

**Published:** 2024-06-13

**Authors:** Ben Madden, Nick Florin

**Affiliations:** Institute for Sustainable Futures, University of Technology Sydney, Ultimo, New South Wales, 2007, Australia

**Keywords:** Packaging, Material flow analysis, Waste management, Circular economy, Separate waste collection, Recycling rates

## Abstract

Household single-use packaging has poor rates of recycling, and presents a challenge in transitioning to a circular packaging economy. This study characterises the flows of household single-use packaging in the municipal waste system for 2020–21 in New South Wales, Australia. Households are an important source of packaging usage in Australia, accounting for over 40 % of all packaging used in 2020–21. Our focus spans 17 single-use packaging materials and 11 formats. We estimate the composition of single-use consumer packaging in the kerbside collection stream, and the ultimate fate of used packaging. Results show 1000 ± 8 % kt of packaging was used by households in NSW in 2020–21 (∼123 kg/cap). Composition of the used packaging stream was dominated by glass (36 %), paper (29 %) and plastic (28 %) packaging. HDPE (26 % of plastic packaging), LDPE (24 %) and PET (19 %) were the main polymers in use. 63 % ± 5 % of used packaging was collected for recycling, and 34 % ± 7 % was recovered via recyclate generation and overseas exports. Glass packaging had the highest recycling rates at 52 % ± 3 %, while plastic packaging had the poorest at 11 % ± 10 %. Findings indicate incorrect disposal of recyclables at the household to mixed-waste systems as a major limitation of the system to improve recycling rates. Expansion in recovery capacity is also essential for improving recycling rates, and the potential for generating the packaging-grade recyclate essential for meeting recycled content targets. The study offers contributions to the understanding of consumer packaging managed within the municipal waste system. Insights gained have application in informing sustainable packaging and waste management strategies.

## Highlights

• Households use more than 40 % of single-use packaging used in New South Wales.
• About 63 % of post-consumer packaging was separately collected, and ∼34 % recycled.
• Post-consumer plastic packaging had the lowest recycling rates at ∼11 %.
• Poor separation behaviour to mixed municipal solid waste is responsible for the largest loss of recyclable material.
• Paper and soft plastic recovery infrastructure capacity expansion necessary.

## 1 Introduction

Increasing consumption of packaging, coupled with low rates of recycling, presents a growing challenge for sustainable waste management globally [[1], [2], [3], [4]]. The circular economy concept has become central for governments worldwide in supporting environmentally sustainable development [5,6], and circular economy initiatives towards packaging sustainability have gained momentum in recent years. The European Union's *Circular Economy Action Plan* [7] and *Packaging and Packaging Waste Regulation* [8] for example underscore the need to transition towards more circular models where packaging materials are reused and recycled efficiently.

Globally, recycling rate targets for circular economy and sustainable packaging initiatives are common [9,10]. Indeed, several countries and regional jurisdictions have implemented voluntary or mandated packaging recycling targets, including 70 % plastic packaging recycling targets for the United Kingdom, Portugal and South Africa [[11], [12], [13]]. In Australia, voluntary *2025 National Packaging Targets* [14] are endorsed by the federal government, and encourage that 70 % of plastic packaging will be recycled or composted; and a 50 % average recycled content in new packaging; to be achieved by 2025. However the packaging sector faces challenges in meeting these ambitious targets, namely in poor rates of collection, and limited recovery infrastructure. The Australian government has committed to reforming packaging regulations, and while consultation is ongoing, anticipated advances include mandatory obligations for packaging design, including minimum recycled content requirements; mandatory extended producer responsibility; and prohibition of harmful chemicals and other contaminants in packaging [15]. These changes necessitate greater resolution around material flows, and scrutiny of the current management system, to understand where system performance can be improved to ensure current and future targets are achievable.

The objective of this study is to characterise the material flows and composition of post-consumer single-use packaging through the municipal waste system in New South Wales, Australia using material flow analysis (MFA). This is a quantitative system modelling approach based on the principles of mass conservation [16], and has been used to investigate household waste flows [[17], [18], [19], [20], [21]]; and flows of used packaging [[22], [23], [24], [25], [26], [27]] at different scales. Whilst many MFA studies focus on plastic packaging, there are few that examine flows of packaging materials other than plastic. There are also limited local studies that model and examine municipal waste systems using MFA with respect to kerbside collection systems. This research helps to address these important research gaps, as well as contributing to the body of work focused specifically on used packaging.

Utilising packaging usage, material recovery, and municipal waste management data, our study aims to estimate the composition of packaging in kerbside mixed waste and dry recyclable fractions. Flows of used packaging are quantified for 15 packaging materials across 11 packaging formats. Performance metrics characterising kerbside diversion, collection and recycling rates are estimated for these packaging types, and compared with the international context and against the 2025 National Packaging Targets.

This study offers valuable contributions to the understanding of household packaging managed within municipal waste systems. Households are an important source of packaging usage, consuming 2.8 million tonnes, or 42 % of all packaging used across Australia in 2020–21 [28]. Greater understanding of the flows of used packaging will help support progress towards sustainable packaging and municipal waste management strategies. Our study establishes a baseline model for household waste flows, from which further analysis and research can be performed, and contributes to the growing global evidence base on the performance of packaging management systems. Insights gained from this work may also have application in informing sustainable packaging principles, including packaging designed for recycling.

## 2 Background

### 2.1 Study area background

New South Wales (NSW) is Australia's most populous state, with 8,097,062 residents as of June 2021 [29]. In 2020–21, waste generated from all sources in NSW was approximately 20.3 million tonnes, compared to 63.4 million tonnes generated nationally [30].

Household waste in NSW is generally disposed to municipally provided bins, usually 120 L or greater in volume. Each bin system is dedicated to specific waste fraction, including mixed waste, dry recyclables and organics. Bins are ‘set-out’ on the kerbside, for collection via specialised waste collection vehicles, with collection occurring weekly for mixed waste and co-collected garden and food waste, and fortnightly for separately collected recyclables including garden waste [31].

**Fig. 1:**
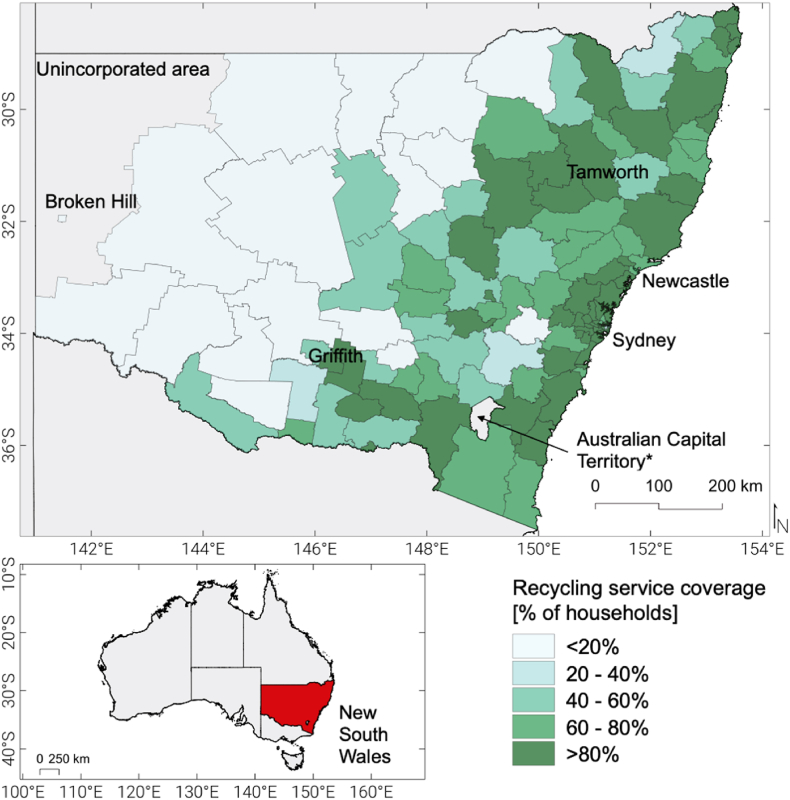
The New South Wales study area, with proportion of households with access to kerbside recycling services shown by local government area, from NSW EPA [31].

Fig. 1 shows the proportion of households with access to a kerbside dry recycling service in NSW by local government area (LGA). Approximately 84 % of NSW households have access to a kerbside dry recyclable service. Generally, areas of significant population (e.g., Sydney metropolitan area) have recycling service coverage above 80 %, however this falls to below 20 % for sparsely populated rural areas of the state.

Table 1 shows the proportion of households in NSW with access to kerbside recycling services for the listed materials, from the NSW waste authority [31]. Glass, paper/paperboard, PET and HDPE plastic, aluminium and steel have the highest coverage, with over 80 % of NSW households having access to kerbside recycling services for these materials. Coverage is also high (77 %) for polymer coated paperboard (PCPB), however as indicated in APCO [32], few recycling pathways exist for PCPB, at least with respect to recovery of material used in packaging. Only 4 % of NSW households have access to a kerbside recycling service that takes polymer film packaging. Only 4 % of households have access to a soft plastics kerbside collection service. Notably, no jurisdictions in NSW currently accepts compostable packaging in municipal recycling fractions. Reasons for this include contamination concerns and limited composting facilities accepting dry compostables [33,34].

**Table 1:** Proportion of households with access to kerbside recycling for key materials, from NSW EPA [31].

Material	% of NSW households with access to kerbside recycling service accepting each material
Glass	84 %
Paper and cardboard	84 %
Plastic - PET, HDPE	84 %
Steel	84 %
Aluminium	82 %
Plastic - LDPE	79 %
Plastic - PP	77 %
Polymer coated paperboard	77 %
Plastic - Other polymers	71 %
Plastic - PS	61 %
Soft plastics	4 %
Compostable packaging	0 %

Approximately 3 million tonnes of total waste was collected from households via the kerbside in 2020–21, with the mixed waste stream accounting for 58 % of all collections. Approximately 51 % of the mixed waste stream is organic waste, primarily kitchen scraps. This limits the recovery opportunities for the mixed waste stream, with mechanical biological treatment (referred locally as AWT, or alternate waste treatment) being the only option available for mixed waste recovery. Approximately 575,000 tonnes of mixed waste sent to AWTs in 2020–21, with 171,900 tonnes recovered [31].

### 2.2 Packaging in New South Wales and Australia

Approximately 6 million tonnes of food, beverage and other retail goods packaging was placed on the market (PoM) in Australia in 2020–21, with NSW packaging accounting for 33 % [28] by mass. Household packaging (i.e., business-to-consumer, used at home) accounted for approximately 46 % of all packaging PoM nationally and in NSW.

Half of all packaging PoM in Australia was fibre-based (55 %), with cardboard packaging making up 42 % of all packaging PoM. A large proportion of fibre-based packaging however is business-to-business packaging, making up 30 % of paper packaging PoM. Table 2 shows overall quantities of household (business-to-consumer) and non-household (business-to-business) packaging placed on the market for Australia, compared with NSW in 2020–21. Approximately 1.2 million tonnes of plastic packaging was PoM, representing 19 % of the overall packaging supply. Low-density (LDPE) and high-density polyethylene (HDPE), and polyethylene terephthalate (PET) had the highest quantities of plastic packaging PoM. Approximately 42 % of all plastic packaging PoM was flexible polymer film packaging, which notably has few recovery pathways locally and internationally, primarily due to high costs associated with recovery of polymer films [35].

**Table 2:** Overall quantities of (household and non-household) packaging placed on the market (PoM) in New South Wales 2020–21, from APCO [28]. Acronyms: PET = polyethylene terephthalate; HDPE = high-density polyethylene; PVC = polyvinyl chloride; LDPE = low-density polyethylene; PP = polypropylene; (E)PS = (expanded) polystyrene.

Packaging material group	Packaging material	Overall packaging PoM in Australia in 2020–21 [tonnes]	Overall packaging PoM in NSW in 2020–21 [tonnes]
Glass	Glass	1,283,000	404,300
Metal	Aluminium	102,000	32,100
	Steel	151,700	45,200
Paper	Boxboard/cartonboard	314,500	98,700
	Corrugated cardboard	2,538,700	874,700
	Polymer coated paperboard	94,100	29,300
	Other fibre packaging	439,400	142,600
Plastic	PET (1)	149,300	48,300
	HDPE (2)	286,900	91,900
	PVC (3)	14,800	4600
	LDPE (4)	331,200	104,100
	PP (5)	215,400	59,500
	(E)PS (6)	46,200	14,500
	Compostable	3800	1200
	Other polymers	131,600	41,400
Total	Total	6,102,600	1,992,400

**Fig. 2:**
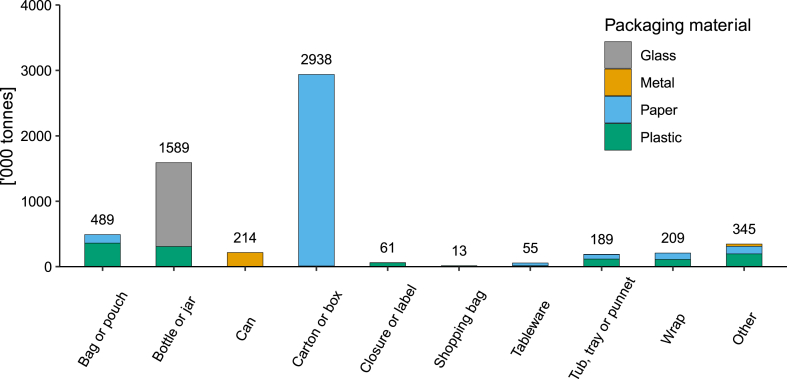
Breakdown of packaging placed on the market in Australia in 2020-21 by packaging format, from APCO [28].

Fig. 2 shows a breakdown of packaging PoM across all of Australia by packaging material group, and format. It was assumed that the breakdown of packaging formats PoM is consistent across all Australian jurisdictions, given similarities in demographic and socioeconomic characteristics. Cartons and boxes made up 48 % of all packaging format types PoM. Bottles and jars made up 26 % as the next highest format, with glass making up 81 % of this category, and the remaining proportion made up of plastic. Plastic bottles and jars were primarily manufactured from HDPE, representing 51 % of all plastic bottles and jars, followed by PET (34 %), and polypropylene, or PP (6 %). Beverage bottles are a particularly important format category, where additional collection pathways exist via NSW's *NSW Return and Earn* scheme [36]. This is a deposit return scheme (DRS), referred to locally as a container deposit scheme (CDS). The NSW CDS is aimed at encouraging recycling and reducing the environmental impact of discarded containers, by providing a financial incentive for disposal (AUD$0.1). Similar deposit return schemes abroad are becoming more widespread for packaging, and have led to significant improvements in bottle collection rates, however comprehensive comparative performance data is limited. Lithuania saw an increase in bottle collection rates from 32 % to 92 % soon after the scheme was implemented [37], and average bottle collection rates across Europe are higher in countries with deposit schemes in place (90.8 %) compared to those without (46.5 %) [38]. Containers collected via dedicated CDS collection channels in NSW bypass kerbside collection and sorting that takes place at material recycling facilities (MRFs), and are in demand by local reprocessors given the relative purity of the stream [28]. Approximately 238,000 tonnes of glass, plastic (PET and HDPE), aluminium and polymer coated paperboard (PCPB) containers placed on the market in NSW in 2020-21 were eligible for refunds via the CDS. Approximately 177,000 tonnes (∼74 %) of these containers were ultimately collected via the CDS.

## 3 Methodology

### 3.1 Scope of analysis

Our analysis is focused on single-use household packaging in NSW that is purchased for and used in the 2020–21 fiscal year. Reusable packaging, making up 4 % of Australian packaging [28], is not in scope. Packaging materials in scope include glass, metal (aluminium and steel), paper (boxboard, cardboard, polymer coated paperboard (PCPB), and other fibres), and plastic (polyethylene terephthalate (PET), high and low density polyethylene (HDPE/LDPE), polyvinyl chloride (PVC), polypropylene (PP), polystyrene (PS/E-PS), compostable polymers, and other polymers). Table 3 summarises material group, type and applicable formats in scope. While we model the flows of individual packaging formats (e.g., PET – bottle or jar; Aluminium – can, etc), we report MFA results on the material, and material group basis.

**Table 3:** Packaging materials in scope for this analysis.

Material group	Material	Formats in scope
Glass	Glass (amber, clear and green)	Bottle or jar; tableware
Metal	Aluminium; steel	Can; closure or label; tubs and trays; tableware; other
Paper	Boxboard/cartonboard; corrugated cardboard; polymer coated paperboard (PCPB); other fibre packaging	Bag or pouch; carton or box; tableware; tubs and trays; wrap; other
Plastic	PET (1); HDPE (2); PVC (3); LDPE (4); PP (5); PS and EPS (6); compostable polymers; other polymers (7)	Bag or pouch; bottle or jar; closure or label; shopping bag; tableware; tubs and trays; wrap; other

**Fig. 3:**
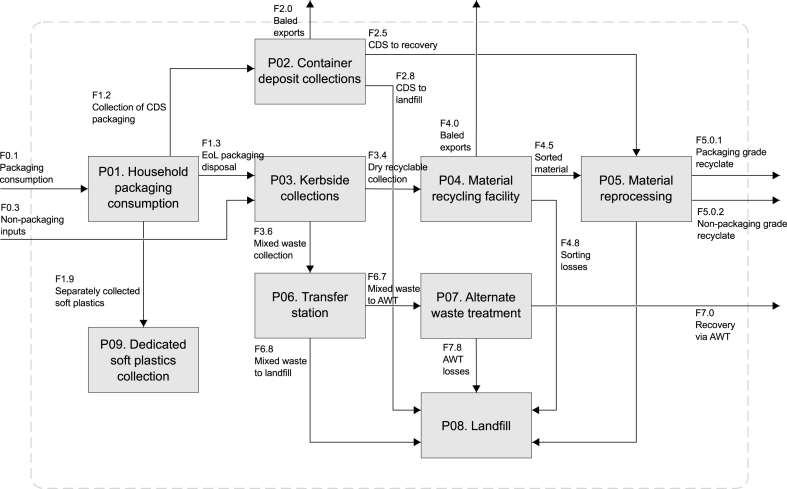
System diagram, showing the material flows between processes considered for this analysis. Material flow and process descriptions are given in the supporting information.

The system investigated is the NSW kerbside municipal waste system (Fig. 3). Processes within our system include a representation of the consumption of packaging within households (process P01 in Fig. 3). End-of-life packaging is disposed of in one of three ways in our system model: via dedicated CDS disposals (process P02), via dedicated soft plastics collection systems (process P09), or via disposal to the kerbside system (process P03).

Eligible containers collected via CDS are transferred to local reprocessing facilities, or exported overseas after baling into single polymer bales. A proportion of containers collected via the CDS system are lost to landfill due to contamination or other system inefficiencies. This is estimated based on life-cycle analysis data on the sorting performance of different waste streams [39]. Dedicated soft plastic collection (P09) was available for the timeframe of analysis via the REDcycle soft plastics recycling service. Consumers could deposit eligible soft plastic items at designated REDcycle bins found in supermarkets across the country. The REDcycle program was decommissioned in November 2022 after it was found the scheme operator was stockpiling collected soft plastics, as recovery pathways for soft plastics are limited [40].

Process P03 represents the kerbside disposal system for household waste. Littering is not included in our system scope, as it is assumed that littered packaging is utilised away from households. The kerbside organic waste collection system is also not included in scope given the negligible quantities of non-packaging material found in the stream [41]. Material collected from the kerbside is transferred to one of three processes: MRFs for sorting (Process P04); AWT facilities (Process P07); or landfill disposal (Process P08). Transfer to both disposal and mixed waste treatment occurs via an intermediate process (Process P06), representing transfer stations. Dry recyclables positively sorted at MRFs are sent to local material reprocessing facilities (Process P05), or are exported overseas for further processing. From reprocessing, material is exported from the system as recyclate for packaging or non-packaging applications. SI Table 1 and SI Table 2 in the Supplementary Information contains further details of processes and flows shown in Fig. 3.

### 3.2 Material flow analysis of household packaging management

The calculation of material flows is derived using a classical Leontief model [42], explained in detail in Bornhöft et al. [43] and Kawecki et al. [44], and summarised here.

External inputs into the system (i.e., F0.1 and F0.3 in Fig. 3) originate from input data, while all other internal and external flows between system processes are determined by transfer coefficients. The transfer coefficient $TC_{i,j}$ represents the proportional mass transfer $q_{i,j}$ from process i to process j, and is a fraction of the cumulative inflow $Q_{i}$ into process i (Eq. (1)):

[1] $$TC_{i,j}=\frac{q_{i,j}}{Q_{i}}$$

Transfer coefficients were derived based on analysis of available Australian packaging usage data and NSW municipal waste data [31]. Unknown transfer coefficients were estimated via back calculation, and all transfer coefficients for process i were normalised (i.e., $\sum_{j}TC_{i,j}=1$), thus $TC_{i,j}\in \left[0,1\right]$. Transfer coefficients used in our analysis can be found in SI Table 3 in the Supplementary Information.

Transfer coefficients can be arranged into the n×n matrix A (Eq. (2)) where n is the total number of system processes, following the method in Bornhöft et al. [43] and Kawecki et al. [44]:

[2] $$A=\left[\begin{array}{cccccc} 1 & \cdots & ‐TC_{1,m} & 0 & \cdots & 0\\ \vdots & \ddots & \vdots & \vdots & \vdots & \vdots \\ ‐TC_{m,1} & \cdots & 1 & 0 & \cdots & 0\\ ‐TC_{m+1,1} & \cdots & ‐TC_{m+1,m} & 1 & \cdots & 0\\ \vdots & \cdots & \vdots & \vdots & \ddots & \vdots \\ ‐TC_{n,1} & \cdots & ‐TC_{n,m} & 0 & \cdots & 1 \end{array}\right]$$

The columns {1,…,m} represent non-accumulating processes, and columns {m+1,…,n} represent accumulating processes including system exports.

The input flows into the system are represented by the column vector G (Eq. (3)), with each element $g_{i}\in G$ representing absolute quantities of materials entering process 1,…,n. For our study, only $g_{1}$ and $g_{3}$ are known (non-zero), representing flows of packaging usage and non-packaging waste inputs respectively (flows F0.1 and F0.3 in Fig. 3). Values for G used in our analysis can be found in SI Table 4 in the Supplementary Information.

[3] $$G=\left[\begin{array}{c} g_{1}\\ g_{2}\\ \vdots \\ g_{n} \end{array}\right]$$

The system equation from Bornhöft et al. [43] (Eq. (4)) contains the unknown column vector X, which represents the mass flow into each process 1,…,n. This equation can be solved for X (Eq. (5)), thereby computing the final inflows into each system process from A and G. All material flows between processes i and j can then be computed from $Q_{i,j}=X_{i}\times TC_{i,j}$.

[4] A∙X=G

[5] $$X=A^{‐1}∙G$$

Table 4 summarises data utilised in this study. Data on packaging flows including PoM in Australia and NSW, as well as proportions of household consumption and recovered packaging nationally was obtained from the *Australian Packaging Covenant Organisation* [28]. Municipal waste data including collections by kerbside system and AWT recovery, was from self-reported local government data [31]. Kerbside audits of the mixed waste [41] and dry recyclable [45] streams were also utilised, in conjunction with quantities of household packaging PoM, to characterise the composition of the household waste stream.

**Table 4:** Data utilised in this analysis.

Data source	Remark
Australian packaging consumption and recovery data, 2020–21 [28]	National level data by material: formats placed on the market; at home packaging consumption; packaging recovery; MSW derived throughput at reprocessing facilities; direct export (bales) NSW specific data: NSW packaging PoM (material only); container deposit scheme flows
Waste avoidance and resource recovery data for NSW, 2020–21 [31]	Data by NSW local government area on waste collected from households for the mixed waste and dry recyclable stream; quantities of dry recyclables and mixed waste recovered/disposed
Analysis of NSW kerbside red lid bin audit data report – results of the 2011–2019 audits [41]	Compositional breakdown of the NSW household mixed waste stream, based on series of kerbside audits performed across NSW
Domestic kerbside waste and recycling in NSW – results of the 2011 waste audits [45]	Compositional breakdown of the NSW dry recyclable stream, based on kerbside audit performed in 2011 and 2014
Article on REDcycle, November 2023 [40]	Quantities of soft plastics collected via the REDcycle scheme in study timeframe

### 3.3 Performance metrics

Key performance metrics for packaging material i are computed, and include the kerbside diversion rate ($KDR_{i}$ in Eq. (6)), collection rate ($CR_{i}$ in Eq. (7)), and recycling rate ($RR_{i}$ in Eq. 8):

[6] $$KDR_{i}=\frac{q_{i}^{dry}}{PoM_{i}}$$

[7] $$CR_{i}=\frac{\left(q_{i}^{dry}+q_{i}^{CDS}+q_{i}^{soft}\right)}{PoM_{i}}$$

[8] $${RR}_{i}=\frac{\left(q_{i}^{bales}+q_{i}^{recyclate}\right)}{{PoM}_{i}}$$

The kerbside diversion rate considers only packaging collected via the dry recyclable kerbside stream (superscript dry). This metric differs to the collection rate, which includes all packaging collected for recycling, including via CDS (superscript CDS) and in-store soft plastics collections (superscript soft). Note that currently AWTs recover only organic material, therefore quantities of household packaging directed to AWT recovery are not considered as collected for recovery. The recycling rate considers the direct export pathway (superscript bales) and local recyclate generation (superscript recyclate).

Comparing collection and recycling rates is convenient for identifying where in the waste management system interventions could be targeted. For example, high recycling rates relative to collection rates indicate that sorting and reprocessing losses are minor, and effort should be placed in improving collection.

### 3.4 Uncertainty analysis

Uncertainty on estimated material flows is evaluated probabilistically based on the method in Laner et al. [46]. Data utilised was qualitatively assessed by giving a score of 1–4 for five separate scoring criteria describing overall data quality: reliability (1), completeness (2), and correlation of the data set with temporal (3), geographical (4) and other factors’ (such as product, technology similarity etc) (5) congruence to the system investigated. Scores in these criteria are used to derive a coefficient of variation (CV) for that dataset, assuming a Gaussian distribution of errors.

Data inputs and transfer coefficients based on actual data are assumed to be Gaussian distributed random values, parameterised by the value used from the data as *η*, and $\sigma^{2}$ from the estimated CV for that dataset. Random values are then drawn from these estimated distributions using Latin-hypercube sampling [47], and used in a Monte Carlo simulation of 10,000 iterations to estimate flow values for the entire system, following approaches in Li et al. [48] and Madden et al. [23].

## 4 Results and discussion

### 4.1 Material flows of post-consumer packaging

**Fig. 4:**
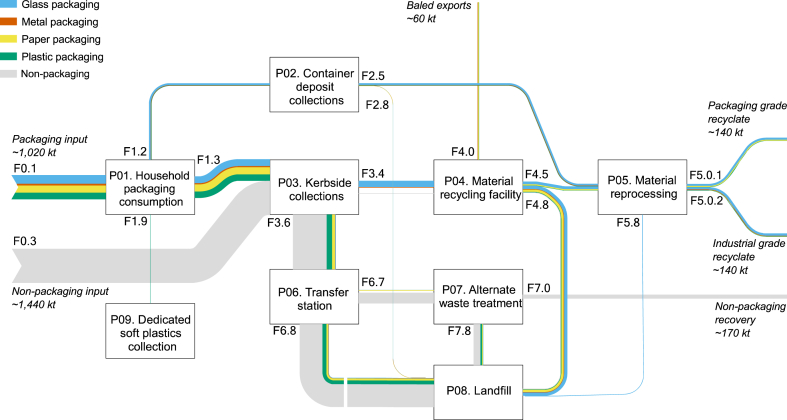
Estimated material flows of the household kerbside system in New South Wales for 2020-21.

Fig. 4 shows a Sankey diagram of our system, aggregated to material group. Exact flow values and uncertainty ranges are provided in the Supplementary Information. Approximately 1,010,000 tonnes of packaging were used by households in NSW in 2020–21. The majority of household packaging (89 %) was disposed to the kerbside system through both the dry recyclable and mixed waste streams. Approximately 109,000 tonnes of packaging, or 11 % of all collections, were collected via the container deposit scheme (CDS) in NSW. Packaging eligible for collection via this pathway included some beverage bottles (glass and PET, with some LDPE and HDPE) and cans (aluminium). Approximately 2300 tonnes of polymer film packaging was collected via dedicated in-store soft plastics collection for stockpiling. Overall, 634,000 tonnes of post-consumer packaging was collected for recycling via kerbside recycling and CDS and in-store soft plastic collection pathways, at a collection rate of 63 %. 338,400 tonnes of this packaging was recovered via recyclate generation or overseas exports at a recycling rate of 34 % of PoM. 669,000 tonnes of packaging was disposed to landfill. Packaging collection and recovery rates are discussed in further detail in Section 4.2.2.

#### 4.1.1 Composition of the kerbside collection stream

**Fig. 5:**
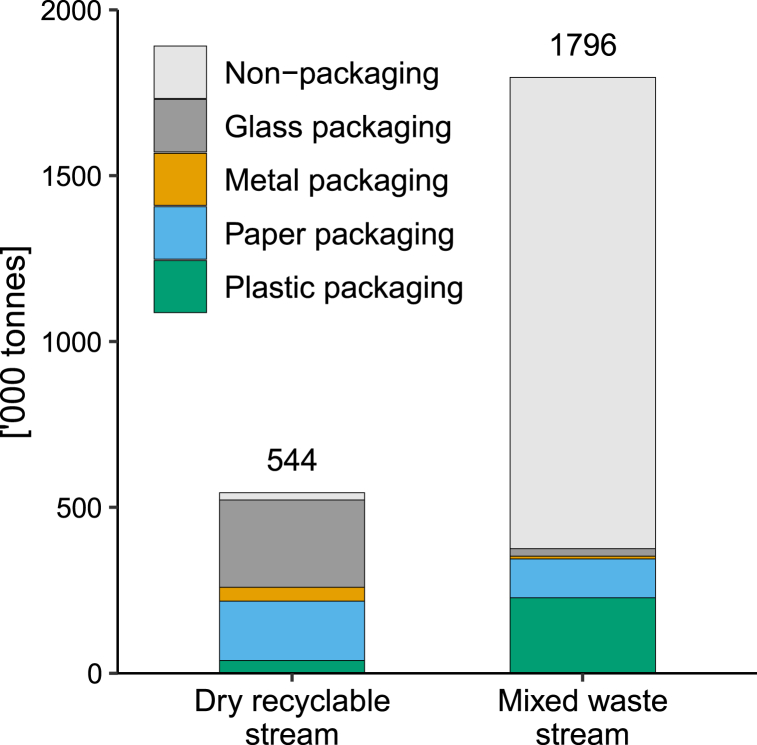
Estimated packaging materials in the New South Wales kerbside system for 2020-21 by quantity.

Fig. 5 shows the composition of the dry recyclable and mixed waste collection streams in terms of packaging and non-packaging materials. Approximately 1.8 million tonnes of household waste was collected via mixed waste kerbside collections, of which 21 % were used packaging material. In comparison, 544,000 tonnes of total waste was collected via the dry recyclable stream, of which 522,500 tonnes was used packaging (96 %). The remainder of the dry recyclable stream consisted of non-packaging material including contamination (4 %). With the growth of e-commerce in recent years leading to an increase in household cardboard box packaging [49], and a continuing decline in print media in Australia [50], the proportion of packaging in the dry recyclable stream has likely increased, as newspaper and other print media would have had a more substantial part of the stream. Ultimately, the dry recyclable stream accounted for 58 % of all used packaging collected via the kerbside.

Kerbside diversion rates are summarised in Table 5 for the packaging materials (on a net basis, i.e., residues and contamination excluded). Overall, the kerbside diversion rate for used packaging was 58 % ± 4 %, and was highest for glass packaging (92 % ± 1 %) and metal packaging (83 % ± 1 %). High diversion rates for these materials might be explained by several factors, including there being well-established recycling systems and separation behaviours for these materials, reducing the likelihood of incorrect disposal.

**Table 5:** Estimated kerbside collections by stream, and estimated diversion rates for packaging materials and formats for NSW, 2020–21. Note uncertainty range on computer diversion rates are relative.

	Collected via dry recyclable collection [tonnes]	Collected via mixed waste collection [tonnes]	Kerbside diversion rate
**Glass**	**264,000 ± 23,100**	**22,800 ± 3500**	**92 % ± 1 %**
**Metal**	**41,300 ± 3700**	**8300 ± 1200**	**83 % ± 1 %**
*Aluminium*	18,600 ± 1700	2800 ± 400	87 % ± 1 %
*Steel*	22,700 ± 2000	5500 ± 800	80 % ± 2 %
**Paper**	**178,400 ± 18,200**	**117,500 ± 14,300**	**60 % ± 4 %**
Boxboard/cartonboard	43,300 ± 4500	28,500 ± 3400	60 % ± 4 %
Corrugated cardboard	105,700 ± 10,700	69,600 ± 8600	60 % ± 4 %
Other fibre packaging	20,800 ± 2100	13,700 ± 1600	60 % ± 6 %
Polymer coated paperboard	8600 ± 900	5700 ± 700	60 % ± 8 %
**Plastic**	**38,800 ± 5500**	**226,800 ± 19,800**	**15 % ± 12 %**
PET	8300 ± 1200	33,300 ± 3000	20 % ± 11 %
HDPE	12,400 ± 1800	59,200 ± 5200	17 % ± 12 %
PVC	0 ± 0	2700 ± 200	0 %
LDPE	4000 ± 500	62,300 ± 5400	6 % ± 12 %
PP	8300 ± 1200	37,100 ± 3300	18 % ± 12 %
PS & EPS	1400 ± 200	4900 ± 400	22 % ± 11 %
Compostable	0 ± 0	400 ± 40	0 %
Other polymers	4400 ± 600	26,900 ± 2300	14 % ± 11 %
**Total packaging**	**522,500 ± 47,600**	**375,400 ± 38,700**	**58 % ± 4 %**

Paper packaging comprised 33 % of the dry recyclable stream, and had a diversion rate of 60 % ± 4 %, despite generally having good technical recyclability. Corrugated cardboard packaging accounted for 59 % of all paper packaging collected via the dry recyclable stream, and boxboard/cartonboard accounted for 24 %. These packaging materials typically have good recyclability, in contrast to polymer coated paperboard (PCPB), which although making up only 5 % of paper packaging collected, has limited pathways for recovery. Other fibre packaging made up 12 % of paper packaging collected via the dry recyclable stream, and is comprised of a mix of recyclable and non-recyclable packaging. Low diversion rates observed may be due to some paper packaging types having poorer recyclability, e.g. high wet-strength carrier board, as well as poor disposal practices from households.

For plastic packaging, the mixed waste stream was the primary pathway for collection, with approximately 227,000 tonnes collected compared to 39,000 tonnes via the dry recyclable stream. This corresponds to a kerbside diversion rate of 15 % ± 7 %. PET, PP and HDPE polymer packaging had the highest diversion rates for plastic packaging at 20 % ± 7 %, 18 % ± 9 % and 17 % ± 6 % respectively, and accounted for a combined 75 % of all plastics collected through the stream.

Approximately 60 % of plastic packaging collected via mixed waste were rigid formats, which typically has good technical recyclability [28], represents a significant loss of potentially recoverable material. While polymer film packaging has poorer technical recyclability, the overall diversion rate for rigid plastic packaging was still poor, at 22 % ± 11 %.

It is likely that improper disposal practices are the key reason for the proportion of recyclable packaging, especially plastics, found in this mixed waste stream. This finding is consistent with other studies examining household waste separation in Austria [51], China [52], and the Netherlands [53]. Note that waste arising from households with no access to a dry recyclables collection system represent only 2 % of the state's total mixed waste collections in 2020–21 [31]. Improving diversion rates for packaging and other recyclables should be a priority for local government decision makers as well as packaging brand owners. The *Australasian Recycling Label (ARL)* [54] exists to help consumers dispose of packaging to the correct kerbside system. However not all plastic packaging features the ARL, and some plastic packaging also does not include information of the polymer type, potentially making correct disposal of plastic packaging to recycling streams difficult. Studies have found that correct sorting and disposal of plastic packaging by consumers at home is perceived as being particularly complex [55], and a major cause for recyclable plastic food packaging being disposed to non-recyclable waste stream [56]. Incentives for households, such as pay-as-you-throw (PAYT) systems, have been shown to improve household recycling and disposal rates [57,58], and given the success of the incentivised CDS system, has promise in NSW. A recent pilot scheme in Australia and NSW led by the *Australian Food and Grocery Council* has explored the implementation of a ‘bag-in-bag’ collection system for kerbside collection of polymer film packaging [59]. A similar program implemented in Catalonia, Spain was assessed in Gibovic et al. [60], with success in improving household diversion rates reported.

#### 4.1.2 Fate of post-consumer packaging

SI Table 5 in the Supplementary Information shows quantities of household packaging material recovered by recycling pathway. Approximately 338,400 tonnes of NSW household packaging material was recycled in 2020–21, with approximately 669,000 tonnes of packaging disposed to landfill. Overall, 34 % ± 2 % of used packaging was recycled. Local processing of used packaging for recyclate accounted for 83 % of all recycling, with 143,300 ± 7 % tonnes of packaging-grade, and 139,100 ± 7 % tonnes of non-packaging grade recyclate produced. 56,000 ± 12 % tonnes of used packaging was exported overseas for further processing after sorting. Paper packaging made up the majority of exports, however from 1 July 2024, paper waste exports will be regulated to ensure a higher quality of export stream, with reduced contamination and mixed paper inputs [61]. To ensure that paper recycling rates remain at a similar level, any regulated paper packaging material not able to be exported must be processed locally. However, paper reprocessing capacity is insufficient to maintain recycling rates from 2020 to 21 into 2024–25 [32].

Significant quantities of plastic packaging were also exported for recovery in adherence with export regulations, ensuring only plastic waste sorted into individual polymers may be eligible for export. Metal packaging was also exported for recycling, however there are no plans to regulate waste metal exports. Local processing capacity is also insufficient to process growing levels of used plastic packaging in the future. Investment in new infrastructure is essential to process growing quantities of used plastic packaging and non-packaging material. There are strong local end-markets for recovered polymers, namely HDPE and PET, which are partly driven by recycled content targets for new packaging. However given the lower quality of recyclate compared to virgin material (especially in the case of polymers), there may be little demand for recycled content in the future without industry incentives or expanded recycled content in new packaging mandates. Investment in further recovery and remanufacturing infrastructure has been identified as a priority in the *NSW Waste and Sustainable Materials Strategy 2041* [62].

**Fig. 6:**
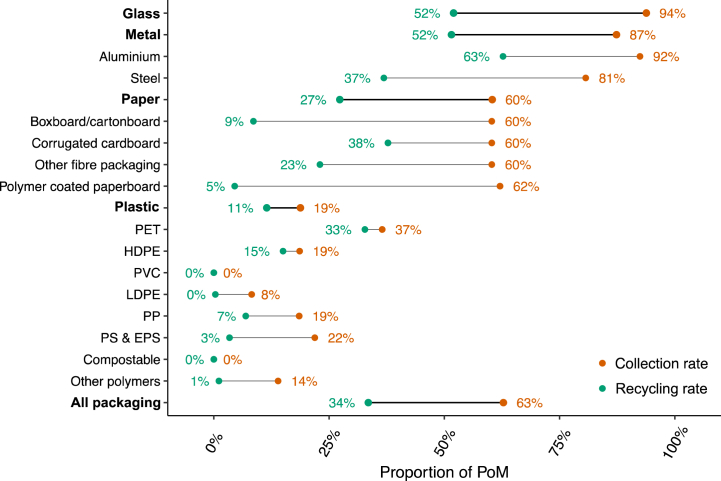
Estimated collection and recycling rates for packaging materials. Aggregated packaging material types (glass, metal, paper, plastic, and all packaging) are bolded. Collection and recycling rates were highest for glass and metal packaging, reflecting high diversion rates observed, with collection rates of 94 % ± 6 % and 87 % ± 6 % respectively, and recycling rates of 52 %±2–3%. Paper packaging had a collection rate of 60 % ± 4 % and a recycling rate of 27 % ± 9 %.

Fig. 6 compares the collection rate and recycling rate for packaging materials. Collection and recycling rates by individual packaging material and format combinations can be found in the Supplementary Information (SI Table 6). The overall collection rate for packaging was 63 % ± 5 %, and the recycling rate was 34 % ± 7 %, indicating overall losses between collection and recycling are approximately 29 % of PoM. These losses can be attributed to sorting losses at MRFs, as well as losses during material reprocessing. These losses are less than collection losses (equal to approximately 37 %), and can be addressed especially with advanced sorting equipment, including high-resolution optical sorting systems which have been shown to increase MRF efficiency [1].

Plastic packaging had the lowest collection and recycling rates, at 19 % ± 7 % and 11 % ± 10 % respectively. Recycling rates for plastic packaging observed in this study are similar, albeit poorer, to rates reported in the literature, especially compared to the EU, where energy recovery and more advanced incentives for better disposal practices are common practices [63]. Antonopoulos et al. [1] for example reports post-consumer plastic packaging recovery of 14 % in the EU27. Van Eygen et al. [27] and Lombardi et al. [22] report a recycling rate for plastic packaging of 26 % in Austria and 40 % in Italy respectively. Note that with 42 % of the plastic packaging stream being polymer films with poor recyclability, the potential plastic packaging recycling rate is limited by the quantity of polymer films, as well as poor collection rates. In the current system, soft plastics are not generally accepted in kerbside recycling, due to there being limited soft plastic recycling pathways in NSW and Australia more generally. This makes AWTs designed to positively sort out plastic packaging from the mixed waste stream (instead of prioritising organic material) a promising pathway given the current system. Indeed, expanding AWT capacity is a priority for the NSW *Waste and sustainable materials strategy 2041*, however with the motivation for improving organic waste recovery from the mixed waste stream. AWT and MBT systems have been shown internationally to potentially improve material recovery from mixed waste streams [64,65]. Chemical recycling and corresponding separate collection of polymer film packaging is another promising pathway for addressing polymer film packaging, and deployment of chemical recycling capacity has also been proposed and approved in NSW and Australia [32]. While chemical recycling does overcome many disadvantages and inefficiencies of mechanical recycling with respect to soft plastics, it is not a mature technology, and implementation is costly [35,66]. Note that some of the costs associated with implementation could be recovered through packaging extended producer responsibility (EPR) schemes, whereby packaging producers take on financial responsibility for collection and/or recycling at end-of-life [[67], [68], [69]].

**Fig. 7:**
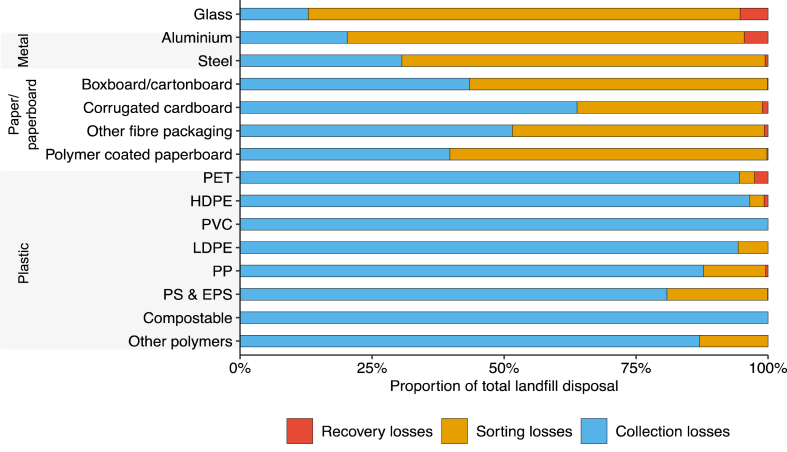
Breakdown of used packaging disposed to landfill in NSW in 2020-21 by source.

Overall, 669,000 tonnes of used packaging was disposed to landfill in NSW in 2020–21. Plastic packaging contributed the most, with approximately 245,000 tonnes of packaging disposed to landfill (37 % of total). Metal packaging had the lowest contribution to overall disposals at 5 %, with approximately 31,700 tonnes of packaging disposed to landfill. Fig. 7 gives a breakdown of used packaging disposed to landfill by source. Total landfill disposal due to collection losses (e.g., incorrect disposal) was approximately 375,400 tonnes, and accounted for 56 % ± 6 % of overall landfill disposals. Collection losses were greatest for the plastic packaging types, consistent with the findings described earlier in Section 4.2.1.

Disposals due to losses at sorting (e.g., due to contamination, sorting inefficiencies) were approximately 280,400 tonnes, accounting for 42 % ± 4 % of all disposals. Sorting losses were greatest for glass packaging (e.g., owing to breakages during transportation and sorting), accounting for 82 % ± 8 % of glass disposals; aluminium (75 % ± 8 % of disposals); and steel (69 % ± 7 % of disposals). Sorting losses for plastic packaging were comparatively smaller, between 3 % and 20 % of overall disposals to landfill.

Sorting losses had the smallest impact on overall disposals for PET, HDPE and PP packaging. Note that sorting efficiency of packaging can be found based on transfer coefficients (i.e., sorting efficiency = $\left(1-TC_{4,8}\right)$), which can be compared to published data on sorting efficiencies. SI Table 7 in the Supplementary Information summarises these efficiencies for plastic packaging, compared to a recent study [1]. Comparison with this data suggests that MRF sorting rates in NSW for key plastic packaging polymers (PET, HDPE, PP) are consistent with those found in the literature. Approximately 90 % of PET, 87 % of HDPE, and 40 % of PP packaging was positively sorted for further reprocessing, comparable to the 81 % ± 11 %, 76 % ± 19 % and 57 % ± 19 % sorting rates for PET, HDPE and PP observed in Antonopoulos et al. [1]. Sorting rates for LDPE were low at approximately 6 %, however the majority of the LDPE packaging stream is polymer film. Although polymer film packaging can be sorted and recovered at relatively high efficiency [70,71], there are no established large-scale recovery processes or markets available for soft plastic recycling in NSW and Australia, and there is little incentive for MRFs to positively sort this material.

### 4.2 Performance against targets

#### 4.2.1 Performance against packaging recycling target

**Fig. 8:**
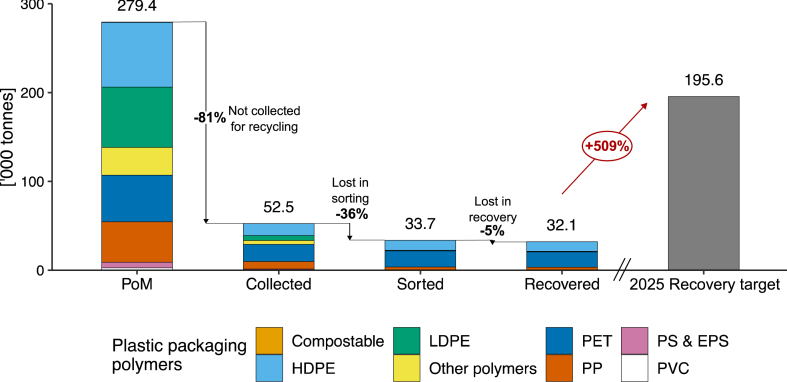
Household plastic packaging polymers placed on the market (PoM), collected for recycling, sorted and recovered in NSW in 2020–21, compared to the 2025 National Packaging Target.

The overall used packaging recycling rate was 34 % ± 7 %, however only plastic packaging has a recycling rate target of 70 % by 2025. Household plastic packaging recycling was significantly below the target, at 11 % ± 10 %. Whilst the target is for all plastic packaging across the country and all sectors, comparison against the national target is helpful, allowing comparison of different systems against a performance benchmark set by national packaging authorities. Fig. 8 shows total plastic packaging PoM, collected, sorted and recovered by polymer, in comparison to the national recycling rate target. The figure illustrates the scale of losses between consumption and collection, and the effort needed in order to match the national level recycling target.

Targeting collection, and substituting non-recyclable plastic packaging for recyclable alternatives, should be prioritised for improving plastic packaging management. Replacing non-recyclable packaging with recyclable options is encouraged under the existing national packaging stewardship scheme [72], however only approximately 25 % of national plastic packaging is considered non-recyclable or of unknown recyclability [28]. Notably in the figure, only PET, HDPE and PP packaging types are recovered in significant quantities, highlighting that material selection is important for improving the recyclability of the plastic packaging stream. Phase-outs of PVC packaging as well as PS and E-PS packaging have already begun nationally, which will likely positively impact recycling rates. Furthermore, planned expansions in polymer film recovery [32] will increase the capacity for locally recovery of flexible packaging, and given that polymer film packaging accounts for approximately 42 % of all plastic packaging, this planned expansion could have a significant impact on improving plastic packaging recovery towards the target.

#### 4.2.2 Performance against packaging recycled content target

The *2025 National Packaging Targets* include recycled content targets (as a proportion of PoM) for all packaging (50 %), all glass (50 %), all metal (35 %), all paper (60 %), and all plastic (20 %) packaging. Sub-material targets also exist for PET (30 %), HDPE (20 %), PP (20 %), and polymer film (10 %) packaging.

Quantities of recycled content found in new packaging PoM in NSW is below target for all packaging material types. For the overall packaging target, a difference of 11 % is observed between the recycled content target, and actual proportions of new packaging with recycled content. Paper packaging has the best performance compared to the target, with a difference of only 7 % observed. Metal packaging had the worst performance compared to the target, with a performance deficiency of 20 %. Recycled aluminium and steel from metal packaging has application across a number of sectors beyond packaging, as secondary metal is highly valued, which may help explain the poor rates of secondary material in metal packaging.

Plastic packaging however had the lowest proportions of recycled content observed, at 3 % for overall plastic packaging. PET packaging had the highest rates of recycled content at 11 %, however still significantly below the PET-specific target of 30 %. Recycled content in HDPE and PP packaging was very low, at 4 % and 3 % respectively. While a polymer film target of 10 % exists, the proportion of recycled content in new polymer film packaging PoM is unknown. With upcoming packaging design and minimum recycled content standards for new packaging [15], greater resolution on recycled content in polymer film packaging should become available.

Recycled content in new packaging comes from either overseas manufactured imports, or local sources. In 2020–21, 51 % of all new packaging PoM in Australia with recycled content was manufactured locally [28]. Some packaging types, including boxboard/cartonboard packaging, and aluminium and steel cans, relied entirely on recycled content sourced from overseas. However for plastic packaging, 93 % of all packaging PoM with recycled content was manufactured locally. Meeting the recycled content target nation-wide for plastic packaging would require increasing imports of recycled packaging manufactured overseas, and/or significant investment in local recovery capacity and infrastructure to improve the quality of recyclate to a standard acceptable for use in new packaging. Meeting the target given current recovery rates (and an assumed 16 % of plastic recovery being of a quality for new packaging [28]) would require an increase in plastic recovery rate of over 500 %. Similar findings were observed in the MFA study of PET management in the EU of Kahlert et al. [37], finding that PET recovery rates would need to more than double in order to meet recycled content targets. Kahlert et al. [37] also suggest that even the adoption of CDS-style systems across the EU for bottles would not result in sufficient change to meet EU recycled content targets, especially as demand for recyclate from other industries drives up the price of available recyclate, and as prices for secondary materials (especially recycled PET) is prone to volatility and fluctuating quality standards [73].

**Fig. 9:**
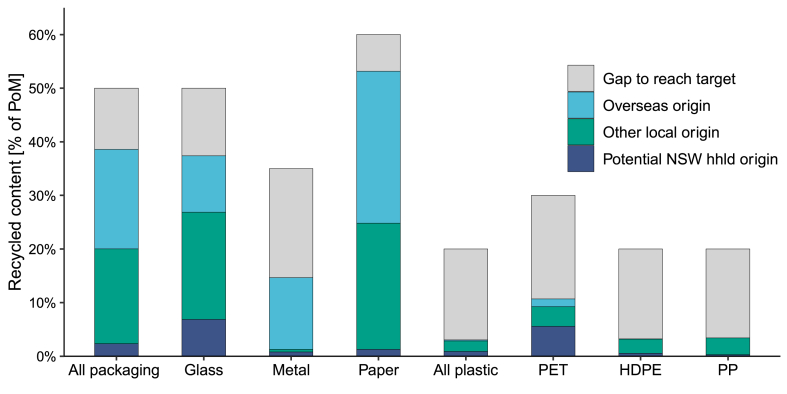
Recycled content in new packaging by origin, compared with potential recycled content generated from NSW household packaging sources. Note hhld = households.

With detailed data on recycled content in household packaging for NSW lacking, packaging grade recyclate produced in NSW can serve as a proxy for estimating the potential quantity of recycled content in the household packaging supply of NSW. Note that in the national dataset, the difference between recycled content placed on the market from post-consumer sources, and national packaging grade recyclate generated, both for 2020–21, was only approximately 6 %, or 2000 tonnes [28]. Fig. 9 includes analysis of recycled content in Australian-wide PoM for the applicable packaging types by origin, with sources of recycled content PoM from: NSW household packaging sources (from this study), originating from other Australian states; and overseas sources; compared with the appropriate recycled content target [28]. Approximately 2 % of national packaging PoM could be derived from recyclate originating from NSW household sources. This proportion was highest for glass (7 %) and PET (6 %). Given that the quantity of NSW household packaging estimated is equivalent to approximately 17 % of all packaging PoM in Australia, this might imply that NSW is underperforming with respect to generating quantities of recycled content PoM. Reasons for this may include a less mature CDS system in NSW (implemented in 2017) compared to other jurisdictions, or a larger quantity of recycled content derived from business-to-business packaging sources in NSW compared to household sources.

### 4.3 Overview of modelling uncertainty

SI Fig. 1 shows the calculated relative uncertainty computed for modelled flows by individual packaging type, and SI Table 8 contains appraisal of data sources used following the method in Laner et al. [46]. Relative uncertainty was highest for non-packaging flows (∼±20 %), expected given data for non-packaging waste flows relied on kerbside audit surveys performed over 10 years before the study timeframe. Packaging flows associated with MRF sorting had high relative uncertainty (∼±16 %), especially for paper and plastic packaging—expected given poorer quality data utilised to characterise MRF sorting processes following the approach in Laner et al. [46]. Flows of polymer film shopping bags collected via the soft-plastics collection program also had high relative uncertainty (∼±23 %) owing to poor data availability on quantities collected via the scheme.

Uncertainty of modelled flow estimates was deemed to be acceptable, and within the range of other MFA studies exploring packaging management (e.g., Van Eygen et al. [27]). Uncertainty could be improved through updated kerbside audit data and MRF sorting data, however such data sets specific to the local context are not available and would require significant primary data collection to obtain.

## 5 Conclusion

Over 1 million tonnes of glass, metal, paper and plastic packaging was consumed by households in NSW in 2020–21. An average packaging collection rate of 63 % ± 5 %, and an average recovery rate of 34 % ± 7 % were observed. Glass packaging types had the highest collection and recovery rates, likely due to maturity of glass collection and recovery in the state. Plastic packaging had the worst performance, especially with respect to polymer film packaging. Without significant system intervention, it is unlikely that the NSW recovery rate for plastic packaging can be improved from 11 % ± 10 % to reach the 70 % performance benchmark. Recycled content targets across the applicable packaging material types will also not likely be met. Moreover, the share of packaging-grade recyclate generated in Australia in 2020-21 from NSW was less than expected, indicating potentially subpar performance of NSW plastic household packaging management from circularity point of view.

This study observed a significant proportion of recyclable packaging in the non-recyclable mixed waste stream from the MFA results, which was highest for plastic packaging. Results indicate that improper disposal of recyclable packaging to the mixed waste stream as a key limiting factor to achieving higher collection and recovery rates for rigid plastics, and packaging material in general. Better packaging design conforming to sustainable packaging guidelines, and improved household awareness and labelling informing best practice disposal methods, may help address this. Comparison of performance against recovery and recycled content targets however underscores a pressing need for expanding local recovery capacity, especially with respect to polymer film packaging.

This work has implications in informing both sustainable packaging practices and MSW strategies. With environment and health impacts being important considerations in the circular economy, future work could look at the potential environmental and human health impacts from moving towards a circular economy for household consumer packaging. These considerations are the subject of future research using the modelling described in this paper.

## Data availability statement

Data will be made available on request.

## CRediT authorship contribution statement

**Ben Madden:** Writing – review & editing, Writing – original draft, Visualization, Methodology, Investigation, Formal analysis, Conceptualization. **Nick Florin:** Writing – review & editing.

## Declaration of competing interest

The authors declare that they have no known competing financial interests or personal relationships that could have appeared to influence the work reported in this paper.
