# Supplementary material for: Characterisation of household single-use packaging flows through a municipal waste system: A material flow analysis for New South Wales, Australia
Source: Heliyon. 2024 Jun 13;10(12):e32878. doi: 10.1016/j.heliyon.2024.e32878 (PMC11226917; doi:10.1016/j.heliyon.2024.e32878)
Supplement: Multimedia component 1 [file mmc1.docx]

**Characterisation of household single-use packaging flows through a municipal waste system: a material flow analysis for New South Wales, Australia**

**Supplementary information**

SI Table 1: System process descriptions

| Process | Description |
| --- | --- |
| P01. Household packaging consumption | Represents the consumption of packaging at the household |
| P02. Container deposit collections | Collection of eligible container deposit scheme (CDS) packaging via dedicated collection pathways (including reverse vending machines, and depots) |
| P03. Kerbside collections | Collection of household waste from the kerbside. Included in scope is the dry recyclable and mixed waste streams |
| P04. Material recycling facility | Represents all material recycling facilities (MRFs) that operate in New South Wales. Material is sorted via mechanical sorting before further processing |
| P05. Material reprocessing | Represents local recovery of packaging material via reprocessing as recyclate |
| P06. Transfer station | Represents transfer stations in New South Wales where waste is aggregated before transfer to further processing or landfilling |
| P07. Alternate waste treatment | Represents alternate waste treatment (AWT) facilities in New South Wales, processing the organic fraction of the mixed waste stream for recovery |
| P08. Landfill | Represents New South Wales landfills. P08 is an accumulating process (stock) |
| P09. Dedicated soft plastics collection | Represents the collection system used for the dedicated separate collection of soft plastic items. The destination of collected plastics in this system are stockpiled |

SI Table 2: System material flow description

| Flow | Description |
| --- | --- |
| F0.1. Packaging consumption | Consumption of household packaging items |
| F0.3. Non-packaging inputs | Consumption of household non-packaging items |
| F1.2. Direct collection of CDS-eligible packaging | Collection of CDS-eligible material |
| F1.3. End-of-life packaging disposal | Packaging disposed to household disposal system |
| F1.9. Separately collected soft plastics | Soft plastics packaging collected via dedicated supermarket collection system |
| F2.5. CDS to recovery | Flows of CDS eligible material sent from the CDS system to local recovery |
| F2.8. CDS to landfill | Losses of CDS eligible material sent to landfill |
| F3.4. Dry recyclable collection | Collection via the kerbside dry recyclable stream |
| F3.6. Mixed waste collection | Collection via the kerbside mixed waste stream |
| F4.0. Baled exports | Exports of sorted and baled material from MRFs |
| F4.5. Sorted material | Material positively sorted at MRFs |
| F4.8. Sorting losses | Losses from MRF sorting |
| F6.7. Mixed waste to AWT | Mixed waste directed to AWT processing for further recovery |
| F6.8. Mixed waste to landfill | Mixed waste sent direct to landfill |
| F7.0. Recovery via AWT | Recovery of mixed waste via AWT (primarily organics) |
| F7.8. AWT losses | Losses of material through the AWT process |
| F5.0.1. Packaging grade recyclate | Recovered material suitable for packaging grade applications |
| F5.0.2. Non-packaging grade recyclate | Recovered material suitable for non-packaging grade applications |
| F5.8. Reprocessing losses | Losses from reprocessing |

SI Table 3: Transfer coefficients used for this study

| Material | Format | F1.2 | F1.3 | F1.13 | F2.8 | F2.5 | F3.4 | F3.6 | F4.9 | F4.8 | F4.5 | F6.8 | F6.7 | F7.12 | F7.8 | F5.10 | F5.11 | F5.8 |
| --- | --- | --- | --- | --- | --- | --- | --- | --- | --- | --- | --- | --- | --- | --- | --- | --- | --- | --- |
| Boxboard | Carton or box | 0 | 1 | 0 | 0 | 0 | 0.6030 | 0.3970 | 0.0957 | 0.8489 | 0.0554 | 0.6770 | 0.3230 | 0 | 1 | 0.6618 | 0.3086 | 0.0296 |
| Boxboard | Tableware | 0 | 1 | 0 | 0 | 0 | 0.6030 | 0.3970 | 0 | 1 | 0 | 0.6770 | 0.3230 | 0 | 1 | 0 | 0 | 0 |
| Corrugated card | Carton or box | 0 | 1 | 0 | 0 | 0 | 0.6030 | 0.3970 | 0.2120 | 0.3624 | 0.4256 | 0.6770 | 0.3230 | 0 | 1 | 0.8048 | 0.1691 | 0.0261 |
| Other fibre | Bag or pouch | 0 | 1 | 0 | 0 | 0 | 0.6030 | 0.3970 | 0.1214 | 0.6185 | 0.2601 | 0.6770 | 0.3230 | 0 | 1 | 0.8075 | 0.1588 | 0.0336 |
| Other fibre | Carton or box | 0 | 1 | 0 | 0 | 0 | 0.6030 | 0.3970 | 0.1213 | 0.6187 | 0.2599 | 0.6770 | 0.3230 | 0 | 1 | 0.8075 | 0.1588 | 0.0336 |
| Other fibre | Other | 0 | 1 | 0 | 0 | 0 | 0.6030 | 0.3970 | 0.1395 | 0.5616 | 0.2989 | 0.6770 | 0.3230 | 0 | 1 | 0.8075 | 0.1588 | 0.0336 |
| Other fibre | Tableware | 0 | 1 | 0 | 0 | 0 | 0.6030 | 0.3970 | 0 | 1 | 0 | 0.6770 | 0.3230 | 0 | 1 | 0 | 0 | 0 |
| Other fibre | Tubs and trays | 0 | 1 | 0 | 0 | 0 | 0.6030 | 0.3970 | 0.1172 | 0.6318 | 0.2510 | 0.6770 | 0.3230 | 0 | 1 | 0.8075 | 0.1588 | 0.0336 |
| Other fibre | Wrap | 0 | 1 | 0 | 0 | 0 | 0.6030 | 0.3970 | 0.1173 | 0.6315 | 0.2512 | 0.6770 | 0.3230 | 0 | 1 | 0.8075 | 0.1588 | 0.0336 |
| PCPB | Carton or box | 0.0644 | 0.9356 | 0 | 0.02 | 0.98 | 0.6030 | 0.3970 | 0.005 | 0.9938 | 0.0009 | 0.6770 | 0.3230 | 0 | 1 | 0.5435 | 0.4077 | 0.0488 |
| PCPB | Other | 0 | 1 | 0 | 0 | 0 | 0.6030 | 0.3970 | 0.005 | 0.9938 | 0.0009 | 0.6770 | 0.3230 | 0 | 1 | 0.5435 | 0.4077 | 0.0488 |
| PCPB | Tableware | 0 | 1 | 0 | 0 | 0 | 0.6030 | 0.3970 | 0.005 | 0.9938 | 0.0009 | 0.6770 | 0.3230 | 0 | 1 | 0.5435 | 0.4077 | 0.0488 |
| PCPB | Tubs and trays | 0 | 1 | 0 | 0 | 0 | 0.6030 | 0.3970 | 0.005 | 0.9938 | 0.0009 | 0.6770 | 0.3230 | 0 | 1 | 0.5435 | 0.4077 | 0.0488 |
| Glass | Bottle or jar | 0.2212 | 0.7787 | 0 | 0.02 | 0.98 | 0.9206 | 0.0794 | 0 | 0.5414 | 0.4586 | 0.6770 | 0.3230 | 0 | 1 | 0.4393 | 0.5139 | 0.0468 |
| Glass | Tableware | 0 | 1 | 0 | 0 | 0 | 0.9206 | 0.0794 | 0 | 1 | 0 | 0.6770 | 0.3230 | 0 | 1 | 0 | 0 | 0 |
| PET | Bag or pouch | 0 | 0.9675 | 0.0325 | 0 | 0 | 0 | 1 | 0 | 0 | 0 | 0.7393 | 0.2607 | 0 | 1 | 0 | 0 | 0 |
| PET | Bottle or jar | 0.2709 | 0.7291 | 0 | 0.02 | 0.98 | 0.2191 | 0.7809 | 0.5804 | 0 | 0.4196 | 0.6770 | 0.3230 | 0 | 1 | 0.6047 | 0.3298 | 0.0656 |
| PET | Other | 0 | 1 | 0 | 0 | 0 | 0.2191 | 0.7809 | 0 | 1 | 0 | 0.6770 | 0.3230 | 0 | 1 | 0 | 0 | 0 |
| PET | Tableware | 0 | 1 | 0 | 0 | 0 | 0.2191 | 0.7809 | 0 | 1 | 0 | 0.6770 | 0.3230 | 0 | 1 | 0 | 0 | 0 |
| PET | Tubs and trays | 0 | 1 | 0 | 0 | 0 | 0.2191 | 0.7809 | 0.5804 | 0 | 0.4196 | 0.6770 | 0.3230 | 0 | 1 | 0.6047 | 0.3298 | 0.0656 |
| PET | Wrap | 0 | 1 | 0 | 0 | 0 | 0 | 1 | 0 | 0 | 0 | 0.7478 | 0.2522 | 0 | 1 | 0 | 0 | 0 |
| HDPE | Bag or pouch | 0 | 0.9675 | 0.0325 | 0 | 0 | 0 | 1 | 0 | 0 | 0 | 0.7393 | 0.2607 | 0 | 1 | 0 | 0 | 0 |
| HDPE | Bottle or jar | 0.0157 | 0.9843 | 0 | 0.02 | 0.98 | 0.2191 | 0.7809 | 0.4953 | 0 | 0.5047 | 0.6770 | 0.3230 | 0 | 1 | 0.2605 | 0.6618 | 0.0777 |
| HDPE | Closure or label | 0 | 1 | 0 | 0 | 0 | 0.2191 | 0.7809 | 0.4953 | 0 | 0.5047 | 0.6770 | 0.3230 | 0 | 1 | 0.2605 | 0.6618 | 0.0777 |
| HDPE | Other | 0 | 1 | 0 | 0 | 0 | 0.2191 | 0.7809 | 0.2739 | 0.4470 | 0.2791 | 0.6770 | 0.3230 | 0 | 1 | 0.2605 | 0.6618 | 0.0777 |
| HDPE | Shopping bag | 0 | 0.9675 | 0.0325 | 0 | 0 | 0 | 1 | 0 | 0 | 0 | 0.7393 | 0.2607 | 0 | 1 | 0 | 0 | 0 |
| HDPE | Wrap | 0 | 1 | 0 | 0 | 0 | 0 | 1 | 0 | 0 | 0 | 0.7478 | 0.2522 | 0 | 1 | 0 | 0 | 0 |
| PVC | Bag or pouch | 0 | 1 | 0 | 0 | 0 | 0 | 1 | 0 | 0 | 0 | 0.6770 | 0.3230 | 0 | 1 | 0 | 0 | 0 |
| PVC | Bottle or jar | 0 | 1 | 0 | 0 | 0 | 0 | 1 | 0 | 0 | 0 | 0.6770 | 0.3230 | 0 | 1 | 0 | 0 | 0 |
| PVC | Closure or label | 0 | 1 | 0 | 0 | 0 | 0 | 1 | 0 | 0 | 0 | 0.6770 | 0.3230 | 0 | 1 | 0 | 0 | 0 |
| PVC | Other | 0 | 1 | 0 | 0 | 0 | 0 | 1 | 0 | 0 | 0 | 0.6770 | 0.3230 | 0 | 1 | 0 | 0 | 0 |
| PVC | Tubs and trays | 0 | 1 | 0 | 0 | 0 | 0 | 1 | 0 | 0 | 0 | 0.6770 | 0.3230 | 0 | 1 | 0 | 0 | 0 |
| PVC | Wrap | 0 | 1 | 0 | 0 | 0 | 0 | 1 | 0 | 0 | 0 | 0.6770 | 0.3230 | 0 | 1 | 0 | 0 | 0 |
| LDPE | Bag or pouch | 0 | 0.9675 | 0.0325 | 0 | 0 | 0 | 1 | 0 | 0 | 0 | 0.7393 | 0.3230 | 0 | 1 | 0 | 0 | 0 |
| LDPE | Bottle or jar | 0 | 1 | 0 | 0 | 0 | 0.2191 | 0.7809 | 0 | 0.9999 | 0 | 0.6770 | 0.3230 | 0 | 1 | 0.0900 | 0.8233 | 0.0867 |
| LDPE | Closure or label | 0 | 1 | 0 | 0 | 0 | 0.2191 | 0.7809 | 0 | 0.9999 | 90 | 0.6770 | 0.3230 | 0 | 1 | 0.0900 | 0.8233 | 0.0867 |
| LDPE | Other | 0 | 1 | 0 | 0 | 0 | 0.2191 | 0.7809 | 0.0014 | 0.9980 | 0.001 | 0.6770 | 0.3230 | 0 | 1 | 0.0900 | 0.8233 | 0.0867 |
| LDPE | Shopping bag | 0 | 0.9675 | 0.0325 | 0 | 0 | 0 | 1 | 0 | 0 | 0 | 0.7393 | 0.2607 | 0 | 1 | 0 | 0 | 0 |
| LDPE | Tableware | 0 | 1 | 0 | 0 | 0 | 0.2191 | 0.7809 | 0 | 1 | 0 | 0.6770 | 0.3230 | 0 | 1 | 0 | 0 | 0 |
| LDPE | Wrap | 0 | 1 | 0 | 0 | 0 | 0.2191 | 0.7809 | 0.0521 | 0.9243 | 0.0235 | 0.6770 | 0.3230 | 0 | 1 | 0.0900 | 0.8233 | 0.0867 |
| PP | Bag or pouch | 0 | 0.9675 | 0.0325 | 0 | 0 | 0 | 1 | 0 | 0 | 0 | 0.7393 | 0.2607 | 0 | 1 | 0 | 0 | 0 |
| PP | Bottle or jar | 0 | 1 | 0 | 0 | 0 | 0.2191 | 0.7809 | 0.2187 | 0.3864 | 0.3949 | 0.6770 | 0.3230 | 0 | 1 | 0.2737 | 0.6367 | 0.0895 |
| PP | Closure or label | 0 | 1 | 0 | 0 | 0 | 0.2191 | 0.7809 | 0.0272 | 0.9236 | 0.0492 | 0.6770 | 0.3230 | 0 | 1 | 0.2737 | 0.6367 | 0.0895 |
| PP | Other | 0 | 1 | 0 | 0 | 0 | 0.2191 | 0.7809 | 0.0558 | 0.8433 | 0.1008 | 0.6770 | 0.3230 | 0 | 1 | 0.2737 | 0.6367 | 0.0895 |
| PP | Tableware | 0 | 1 | 0 | 0 | 0 | 0.2191 | 0.7809 | 0 | 1 | 0 | 0.6770 | 0.3230 | 0 | 1 | 0 | 0 | 0 |
| PP | Tubs and trays | 0 | 1 | 0 | 0 | 0 | 0.2191 | 0.7809 | 0.2119 | 0.4056 | 0.3825 | 0.6770 | 0.3230 | 0 | 1 | 0.2737 | 0.6367 | 0.0895 |
| PP | Wrap | 0 | 1 | 0 | 0 | 0 | 0 | 1 | 0 | 0 | 0 | 0.7478 | 0.2522 | 0 | 1 | 0 | 0 | 0 |
| PS & EPS | Bottle or jar | 0 | 1 | 0 | 0 | 0 | 0.2191 | 0.7809 | 0.0362 | 0.8851 | 0.0787 | 0.6770 | 0.3230 | 0 | 1 | 0.2737 | 0.9528 | 0.0247 |
| PS & EPS | Carton or box | 0 | 1 | 0 | 0 | 0 | 0.2191 | 0.7809 | 0.0603 | 0.8087 | 0.1310 | 0.6770 | 0.3230 | 0 | 1 | 0.2737 | 0.9528 | 0.0247 |
| PS & EPS | Closure or label | 0 | 1 | 0 | 0 | 0 | 0.2191 | 0.7809 | 0.0362 | 0.8851 | 0.0787 | 0.6770 | 0.3230 | 0 | 1 | 0.2737 | 0.9528 | 0.0247 |
| PS & EPS | Other | 0 | 1 | 0 | 0 | 0 | 0.2191 | 0.7809 | 0.0805 | 0.7445 | 0.1750 | 0.6770 | 0.3230 | 0 | 1 | 0.2737 | 0.9528 | 0.0247 |
| PS & EPS | Tableware | 0 | 1 | 0 | 0 | 0 | 0.2191 | 0.7809 | 0 | 1 | 0 | 0.6770 | 0.3230 | 0 | 1 | 0 | 0 | 0 |
| PS & EPS | Tubs and trays | 0 | 1 | 0 | 0 | 0 | 0.2191 | 0.7809 | 0.0286 | 0.9091 | 0.0623 | 0.6770 | 0.3230 | 0 | 1 | 0.2737 | 0.9528 | 0.0247 |
| Compostable | Bag or pouch | 0 | 1 | 0 | 0 | 0 | 0 | 1 | 0 | 0 | 0 | 0.6770 | 0.3230 | 0 | 1 | 0 | 0 | 0 |
| Compostable | Shopping bag | 0 | 1 | 0 | 0 | 0 | 0 | 1 | 0 | 0 | 0 | 0.6770 | 0.3230 | 0 | 1 | 0 | 0 | 0 |
| Compostable | Tableware | 0 | 1 | 0 | 0 | 0 | 0 | 1 | 0 | 0 | 0 | 0.6770 | 0.3230 | 0 | 1 | 0 | 0 | 0 |
| Other polymers | Bag or pouch | 0 | 1 | 0 | 0 | 0 | 0 | 1 | 0 | 0 | 0 | 0.7478 | 0.2522 | 0 | 1 | 0 | 0 | 0 |
| Other polymers | Bottle or jar | 0 | 1 | 0 | 0 | 0 | 0.2191 | 0.7809 | 0.001 | 0.9738 | 0.0250 | 0.6770 | 0.3230 | 0 | 1 | 0 | 0.9862 | 0.0138 |
| Other polymers | Closure or label | 0 | 1 | 0 | 0 | 0 | 0.2191 | 0.7809 | 0 | 1 | 0 | 0.6770 | 0.3230 | 0 | 1 | 0 | 0 | 0 |
| Other polymers | Other | 0 | 1 | 0 | 0 | 0 | 0.2191 | 0.7809 | 0.008 | 0.8190 | 0.1727 | 0.6770 | 0.3230 | 0 | 1 | 0 | 0.9862 | 0.0138 |
| Other polymers | Tableware | 0 | 1 | 0 | 0 | 0 | 0.2191 | 0.7809 | 0 | 1 | 0 | 0.6770 | 0.3230 | 0 | 1 | 0 | 0 | 0 |
| Other polymers | Wrap | 0 | 1 | 0 | 0 | 0 | 0 | 1 | 0 | 0 | 0 | 0.7478 | 0.2522 | 0 | 1 | 0 | 0 | 0 |
| Aluminium | Can | 0.4311 | 0.5689 | 0 | 0.0200 | 0.9800 | 0.8691 | 0.1309 | 0.4727 | 0.5250 | 0.0023 | 0.6770 | 0.3230 | 0 | 1 | 0.1243 | 0.8351 | 0.0405 |
| Aluminium | Closure or label | 0 | 1 | 0 | 0 | 0 | 0.8691 | 0.1309 | 0 | 1 | 0 | 0.6770 | 0.3230 | 0 | 1 | 0 | 0 | 0 |
| Aluminium | Other | 0 | 1 | 0 | 0 | 0 | 0.8691 | 0.1309 | 0 | 1 | 0 | 0.6770 | 0.3230 | 0 | 1 | 0 | 0 | 0 |
| Aluminium | Tubs and trays | 0 | 1 | 0 | 0 | 0 | 0.8691 | 0.1309 | 0 | 1 | 0 | 0.6770 | 0.3230 | 0 | 1 | 0 | 0 | 0 |
| Steel | Can | 0.0065 | 0.9935 | 0 | 0.0200 | 0.9800 | 0.8057 | 0.1943 | 0.3202 | 0.5471 | 0.1327 | 0.6770 | 0.3230 | 0 | 1 | 0.0172 | 0.9506 | 0.0323 |
| Steel | Closure or label | 0 | 1 | 0 | 0 | 0 | 0.8057 | 0.1943 | 0 | 1 | 0 | 0.6770 | 0.3230 | 0 | 1 | 0 | 0 | 0 |
| Steel | Other | 0 | 1 | 0 | 0 | 0 | 0.8057 | 0.1943 | 0.3612 | 0.4892 | 0.1497 | 0.6770 | 0.3230 | 0 | 1 | 0.0172 | 0.9506 | 0.0323 |
| Steel | Tableware | 0 | 1 | 0 | 0 | 0 | 0.8057 | 0.1943 | 0 | 1 | 0 | 0.6770 | 0.3230 | 0 | 1 | 0 | 0 | 0 |
| Organics | Non-packaging | 0 | 0 | 0 | 0 | 0 | 0.0136 | 0.9864 | 0 | 1 | 0 | 0.6770 | 0.3230 | 0.5888 | 0.4112 | 0 | 0 | 0 |
| Non-packaging | Non-packaging | 0 | 0 | 0 | 0 | 0 | 0.0178 | 0.9822 | 0 | 1 | 0 | 0.6770 | 0.3230 | 0 | 1 | 0 | 0 | 0 |

SI Table 4: Input values used for the material flow analysis performed in this study

| Material | Format | F0.1 [tonnes] | F0.3 [tonnes] |
| --- | --- | --- | --- |
| Boxboard/cartonboard | Carton or box | 68,509±8% | 0±0% |
| Boxboard/cartonboard | Tableware | 3,243±8% | 0±0% |
| Corrugated cardboard | Carton or box | 175,256±8% | 0±0% |
| Other fibre packaging | Bag or pouch | 10,142±8% | 0±0% |
| Other fibre packaging | Carton or box | 2,020±8% | 0±0% |
| Other fibre packaging | Other | 9,128±8% | 0±0% |
| Other fibre packaging | Tableware | 81±8% | 0±0% |
| Other fibre packaging | Tub, tray or punnet | 5,152±8% | 0±0% |
| Other fibre packaging | Wrap | 7,893±8% | 0±0% |
| Polymer coated paperboard | Carton or box | 10,421±8% | 0±0% |
| Polymer coated paperboard | Other | 92±8% | 0±0% |
| Polymer coated paperboard | Tableware | 4,010±8% | 0±0% |
| Polymer coated paperboard | Tub, tray or punnet | 456±8% | 0±0% |
| Glass | Bottle or jar | 368,296±8% | 0±0% |
| Glass | Tableware | 18±8% | 0±0% |
| PET | Bag or pouch | 1325±8% | 0±0% |
| PET | Bottle or jar | 39,865±8% | 0±0% |
| PET | Other | 3271±8% | 0±0% |
| PET | Tableware | 419±8% | 0±0% |
| PET | Tub, tray or punnet | 5331±8% | 0±0% |
| PET | Wrap | 2,320±8% | 0±0% |
| HDPE | Bag or pouch | 14,422±8% | 0±0% |
| HDPE | Bottle or jar | 40,010±8% | 0±0% |
| HDPE | Closure or label | 640±8% | 0±0% |
| HDPE | Other | 16,712±8% | 0±0% |
| HDPE | Shopping bag | 898±8% | 0±0% |
| HDPE | Wrap | 105±8% | 0±0% |
| PVC | Bag or pouch | 187±8% | 0±0% |
| PVC | Bottle or jar | 505±8% | 0±0% |
| PVC | Closure or label | 114±8% | 0±0% |
| PVC | Other | 270±8% | 0±0% |
| PVC | Tub, tray or punnet | 114±8% | 0±0% |
| PVC | Wrap | 1,473±8% | 0±0% |
| LDPE | Bag or pouch | 47,873±8% | 0±0% |
| LDPE | Bottle or jar | 1,575±8% | 0±0% |
| LDPE | Closure or label | 224±9% | 0±0% |
| LDPE | Other | 1,954±8% | 0±0% |
| LDPE | Shopping bag | 1,928±8% | 0±0% |
| LDPE | Tableware | 2±8% | 0±0% |
| LDPE | Wrap | 14,357±8% | 0±0% |
| PP | Bag or pouch | 3691±8% | 0±0% |
| PP | Bottle or jar | 3,722±8% | 0±0% |
| PP | Closure or label | 4,461±8% | 0±0% |
| PP | Other | 11,118±8% | 0±0% |
| PP | Tableware | 269±8% | 0±0% |
| PP | Tub, tray or punnet | 18,405±8% | 0±0% |
| PP | Wrap | 3,899±8% | 0±0% |
| PS & EPS | Bottle or jar | 17±8% | 0±0% |
| PS & EPS | Carton or box | 1,248±8% | 0±0% |
| PS & EPS | Closure or label | 100±8% | 0±0% |
| PS & EPS | Other | 2,385±8% | 0±0% |
| PS & EPS | Tableware | 1,087±8% | 0±0% |
| PS & EPS | Tub, tray or punnet | 1,436±8% | 0±0% |
| Compostable | Bag or pouch | 122±8% | 0±0% |
| Compostable | Shopping bag | 31±8% | 0±0% |
| Compostable | Tableware | 273±8% | 0±0% |
| Other polymers | Bag or pouch | 10,600±8% | 0±0% |
| Other polymers | Bottle or jar | 4120±8% | 0±0% |
| Other polymers | Closure or label | 7,606±8% | 0±0% |
| Other polymers | Other | 8,175±8% | 0±0% |
| Other polymers | Tableware | 4±8% | 0±0% |
| Other polymers | Wrap | 763±8% | 0±0% |
| Aluminium | Can | 36,393±8% | 0±0% |
| Aluminium | Closure or label | 2±8% | 0±0% |
| Aluminium | Other | 55±8% | 0±0% |
| Aluminium | Tub, tray or punnet | 683±8% | 0±0% |
| Steel | Can | 21,541±8% | 0±0% |
| Steel | Closure or label | 454±8% | 0±0% |
| Steel | Other | 6,337±8% | 0±0% |
| Steel | Tableware | 3±8% | 0±0% |
| Organics | Non-packaging | 0±0% | 916,322±10% |
| Other non-packaging | Non-packaging | 0±0% | 526,303±10% |

SI Table 5: Estimated household packaging recovery pathways in New South Wales for 2020-21. Note quantities are rounded to 2 significant figures

| Packaging material | Baled overseas exports [tonnes] | Locally produced non-packaging grade recyclate [tonnes] | Locally produced packaging grade recyclate [tonnes] |
| --- | --- | --- | --- |
| Glass | 0 ± 0 | 103,300 ± 10,500 | 88,300 ± 9,400 |
| Metal | 15,900 ± 1,800 | 15,900 ± 1,600 | 2,000 ± 300 |
| *Aluminium* | *8,500 ± 1,000* | *12,900 ± 1,200* | *2,000 ± 200* |
| *Steel* | *7,400 ± 800* | *3,000 ± 300* | *<100 ± 10* |
| Paper | 29,000 ± 4,200 | 9,500 ± 1,400 | 42,500 ± 5,600 |
| *Boxboard/carton* | *4,000 ± 600* | *700 ± 100* | *1,500 ± 200* |
| *Corrugated card.* | *22,400 ± 2,600* | *7,600 ± 1,000* | *36,300 ± 4,100* |
| *Polymer coated paperboard* | *<100 ± 8* | *300 ± 40* | *400 ± 50* |
| *Other fibre* | *2,600 ± 300* | *900 ± 100* | *4,500 ± 500* |
| Plastic | 11,200 ± 1,900 | 10,500 ± 1,700 | 10,500 ± 1,800 |
| *PET* | *4,400 ± 600* | *4,500 ± 600* | *8,300 ± 1,000* |
| *HDPE* | *5,300 ± 800* | *4,000 ± 600* | *1,600 ± 200* |
| *LDPE* | *200 ± 30* | *<100 ± 10* | *<100 ± 1* |
| *PP* | *1,200 ± 200* | *1,400 ± 200* | *600 ± 100* |
| *PVC* | *0 ± 0* | *0 ± 0* | *0 ± 0* |
| *PS & EPS* | *<100 ± 10* | *100 ± 20* | *0 ± 0* |
| *Compostable* | *0 ± 0* | *0 ± 0* | *0 ± 0* |
| *Other polymers* | *<100 ± 5* | *300 ± 60* | *0 ± 0* |
| Total | 56,000 ± 7,900 | 139,100 ± 15,200 | 143,300 ± 17,100 |

SI Table 6: Estimated packaging material and format collection and recovery rates

| Material | Format | Collection rate | Recovery rate |
| --- | --- | --- | --- |
| Boxboard | Carton or box | 60% | 9% |
| Boxboard | Tableware | 60% | 0% |
| Corrugated cardboard | Carton or box | 60% | 38% |
| Other fibre | Bag or pouch | 60% | 22% |
| Other fibre | Carton or box | 60% | 22% |
| Other fibre | Other | 60% | 26% |
| Other fibre | Tableware | 60% | 0% |
| Other fibre | Tubs and trays | 60% | 22% |
| Other fibre | Wrap | 60% | 22% |
| PCPB | Carton or box | 63% | 6% |
| PCPB | Other | 60% | 0% |
| PCPB | Tableware | 60% | 0% |
| PCPB | Tubs and trays | 60% | 0% |
| Glass | Bottle or jar | 94% | 52% |
| Glass | Tableware | 92% | 0% |
| PET | Bag or pouch | 3% | 0% |
| PET | Bottle or jar | 43% | 40% |
| PET | Other | 22% | 0% |
| PET | Tableware | 22% | 0% |
| PET | Tubs and trays | 22% | 21% |
| PET | Wrap | 0% | 0% |
| HDPE | Bag or pouch | 3% | 0% |
| HDPE | Bottle or jar | 23% | 22% |
| HDPE | Closure or label | 22% | 21% |
| HDPE | Other | 22% | 12% |
| HDPE | Shopping bag | 3% | 0% |
| HDPE | Wrap | 0% | 0% |
| PVC | Bag or pouch | 0% | 0% |
| PVC | Bottle or jar | 0% | 0% |
| PVC | Closure or label | 0% | 0% |
| PVC | Other | 0% | 0% |
| PVC | Tubs and trays | 0% | 0% |
| PVC | Wrap | 0% | 0% |
| LDPE | Bag or pouch | 3% | 0% |
| LDPE | Bottle or jar | 22% | 0% |
| LDPE | Closure or label | 22% | 0% |
| LDPE | Other | 22% | 0% |
| LDPE | Shopping bag | 3% | 0% |
| LDPE | Tableware | 22% | 0% |
| LDPE | Wrap | 22% | 2% |
| PP | Bag or pouch | 3% | 0% |
| PP | Bottle or jar | 22% | 13% |
| PP | Closure or label | 22% | 2% |
| PP | Other | 22% | 3% |
| PP | Tableware | 22% | 0% |
| PP | Tubs and trays | 22% | 12% |
| PP | Wrap | 0% | 0% |
| PS & EPS | Bottle or jar | 22% | 2% |
| PS & EPS | Carton or box | 22% | 4% |
| PS & EPS | Closure or label | 22% | 2% |
| PS & EPS | Other | 22% | 6% |
| PS & EPS | Tableware | 22% | 0% |
| PS & EPS | Tubs and trays | 22% | 2% |
| Compostable | Bag or pouch | 0% | 0% |
| Compostable | Shopping bag | 0% | 0% |
| Compostable | Tableware | 0% | 0% |
| Other polymers | Bag or pouch | 0% | 0% |
| Other polymers | Bottle or jar | 22% | 1% |
| Other polymers | Closure or label | 22% | 0% |
| Other polymers | Other | 22% | 4% |
| Other polymers | Tableware | 22% | 0% |
| Other polymers | Wrap | 0% | 0% |
| Aluminium | Can | 93% | 64% |
| Aluminium | Closure or label | 87% | 0% |
| Aluminium | Other | 87% | 0% |
| Aluminium | Tubs and trays | 87% | 0% |
| Steel | Can | 81% | 37% |
| Steel | Closure or label | 81% | 0% |
| Steel | Other | 81% | 41% |
| Steel | Tableware | 81% | 0% |
| Organics | Non-packaging | 1% | 19% |
| Non-packaging | Non-packaging | 2% | 0% |

SI Table 7: Estimated material recycling facility sorting rates for household plastic packaging by polymer type. Note the ±% value is relative, not absolute

| Plastic packaging polymer | Estimated MRF sorting rate from this study | Average MRF sorting rate in Antonopoulos et al. (2021) |
| --- | --- | --- |
| PET | 90%±14% | 81%±11% |
| HDPE | 87%±15% | 76%±19% |
| LDPE | 6%±18% | NA |
| PP | 40%±16% | 57%±18% |
| PVC | 0%±0% | 73%±NA |
| PS & EPS | 16%±17% | 47%±12% |
| Compostable | 0%±0% | NA |
| Other polymers | 8%±18% | NA |

SI Table 8: Characterisation of data uncertainty, based on Laner et al. 2016

| Data point | Reliability score | Complete-ness score | Temporal correl. score | Geo. correl. score | Other correl. score | Remark |
| --- | --- | --- | --- | --- | --- | --- |
| NSW packaging PoM (APCO, 2023a) | 2 | 1 | 1 | 1 | 1 | Data is complete, however generation process is survey therefore not 100% reliable |
| Australia business-to-consumer (at-home) packaging consumption (APCO, 2023a) | 2 | 1 | 1 | 2 | 1 | Data is for all Australia therefore geographic correlation is lesser |
| CDS eligible packaging PoM and redeemed in NSW (APCO, 2023a) | 2 | 1 | 1 | 1 | 1 | Data is complete, however generation process is survey therefore not 100% reliable |
| Australian packaging recovery data (APCO, 2023a) | 2 | 1 | 1 | 1 | 1 | Data is complete, however generation process is survey there not 100% reliable |
| NSW municipal waste and resource recovery data for 2020-21 (NSW EPA, 2023) | 1 | 2 | 1 | 1 | 2 | Government source. Completeness score due to differences in material aggregation. Other correlation score due to not being packaging specific |
| Mixed waste kerbside bin audit (Rawtec, 2020) | 1 | 2 | 2 | 1 | 2 | Based on survey with full sampling methodology provided. Survey conducted in 2019. Completeness and other correlation score due to not being packaging specific |
| Dry recyclable kerbside bin audit (NSW EPA, 2014b) | 1 | 2 | 4 | 1 | 2 | Based on survey will full sampling methodology explained, however the survey was conducted in 2011 and reported in 2014 |
| Data on REDcycle collection (Miles, 2023) | 3 | 3 | 1 | 3 | 1 | Data is from a reputable media source, however data source is not explained, and data is aggregated for all packaging types collected via the scheme across Australia |

SI Figure 1: Estimated relative uncertainty of calculated material flows by packaging type
